# *In vivo* and *ex vivo* dermoscopy of lesions from implantation of human papillomavirus in tattoos: report of two cases^☆^^☆☆^

**DOI:** 10.1016/j.abd.2019.02.008

**Published:** 2019-12-17

**Authors:** John Verrinder Veasey, Ana Luisa Nasser Erthal, Rute Facchini Lellis

**Affiliations:** a Dermatology Clinic, Irmandade da Santa Casa de Misericórdia de São Paulo, São Paulo, SP, Brazil; b Pathology Laboratory, Irmandade da Santa Casa de Misericórdia de São Paulo, São Paulo, SP, Brazil

**Keywords:** Dermoscopy, Histology, Comparative, Tattooing, Warts

## Abstract

The number of individuals with tattoos has been increasing worldwide, alongside with reports of complications varying from reactions to the injected pigments to infections caused by agents inoculated in the pigmentation process. The diagnosis of such unwanted events can be obtained through complementary non-invasive methods, preserving the maximum of the tattoo design. The authors present two cases of patients with warts on tattooing, and correlate their clinical aspects to *in vivo* and *ex vivo* dermoscopy, and to the findings in the histopathological examination, aiming to determine patterns that aid the diagnosis of these lesions without performing biopsy.

The practice of skin ornamentation is a habit as old as human civilization, having been found in mummies from the period between 2000 and 4000 BC.^1^ Currently, it is estimated that 21% of the adult population of the United States has at least one tattoo,^2^ and 25% in Germany.^3^ Regarding the Brazilian population, the prevalence of individuals with a tattoo varies from 10% to 26% in men and from 10% to 22% in women.^4^

During the tattooing process, the individual is exposed to histological reactions to the pigments (eczematous, sarcoid, granulomatous, pseudolymphomatous),^4^ as well as infections caused by different pathogens, which can lead to severe sequelae and difficult treatment.^1^, ^2^ Among these infections, there are reports of systemic diseases such as hepatitis B, hepatitis C, and HIV infection,^1^ and those restricted to the skin, such as atypical mycobacterioses^2^, ^5^ and warts.^6^

This report presents two cases of patients who, after receiving a tattoo, developed lesions on the pigmentation line, having a clinical, dermatoscopic, and histopathological diagnosis of wart.

In the first case, a 39-year-old man with a tattoo on the left side of the left calf for eight years reported localized lesions on the tattoo for seven years. He denied continued use of medications or use of topical medications on the lesions. At the examination, there were numerous erythematous scaling papules that varied between 3 and 7 mm in diameter, arranged on the contours of the tattoo, forming linear paths. At dermoscopy, some projections of similar diameter and length were observed in a “knob” pattern over the papules, with thrombosed glomerular capillaries, giving the appearance of red spots on the lesion's surface. An incisional biopsy with punch was performed and an *ex vivo* dermoscopy was performed for a better analysis of the cutaneous structures and their alterations. A papillomatous verruciform appearance in the epidermis was presented, with underlying pigment in the dermis (Fig. 1). The result of histopathological examination was compatible to a wart over a tattoo (Fig. 2).

**Figure 1:**
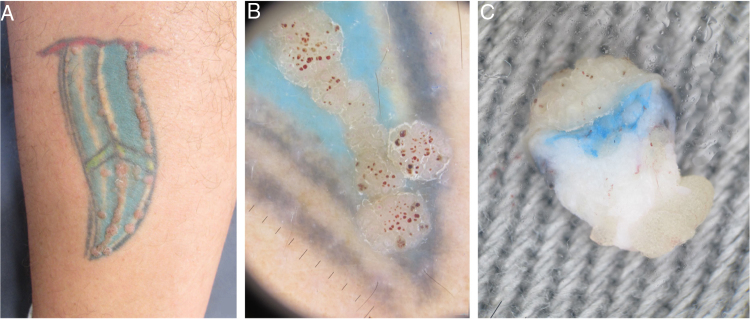
(A) Clinical aspect of the tattoo with multiple papules arranged linearly. (B) *In vivo* dermoscopy (×10 magnification) showing papules with digitiform and red dotted projections corresponding to vascular ectasias. (C) *Ex vivo* dermoscopy (×20 magnification) showing papillomatous and dotted hemorrhagic projections in the epidermis, with presence of pigment in the superficial dermis.

**Figure 2:**
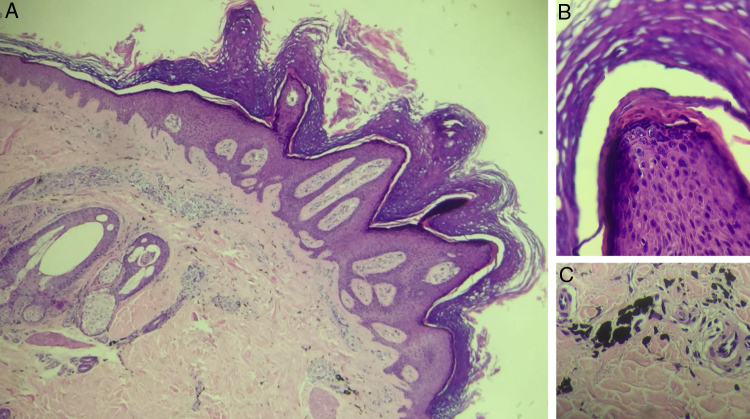
Histopathological exam of patient 1, stained with hematoxylin & eosin. (A) ×40 magnification, presenting epidermis with papillomatosis, prominent hyperkeratosis with parakeratosis, hypergranulosis, acanthosis and elongated epidermal ridges, and dermis with presence of extracellular deposits of black pigment, compatible with exogenous pigment. (B) ×200 magnification evidencing detail of papillomatosis. (C) ×200 magnification with detail of black pigment accumulation in dermis.

In the second case, a 33-year-old man with a tattoo on the left upper limb for 12 years reported lesions that disseminated six years previously on the design of his tattoo. He was positive for HIV infection, using zidovudine, lamivudine, ritonavir, and darunavir (viral load undetectable and CD4+ cells count: 582/mm^3^) and genital condylomata acuminate (multiple papillomatous warts), under treatment. Physical examination showed a dermatosis located on the left arm characterized by multiple flattened erythematous and confluent papules, forming plaques with defined and irregular borders. Some lesions accompanied the tattoo design, while others affected healthy skin. Dermoscopy presented a nonspecific appearance, with isolated spots of desquamation. An incisional biopsy was performed, and as in the first case, an *ex vivo* dermoscopy of was performed, showing a slightly thickened epidermis with discrete papillomatosis and presence of pigment in the dermis (Fig. 3). The result of the histopathological examination confirmed the diagnosis of a wart over a tattoo (Fig. 4).

**Figure 3:**
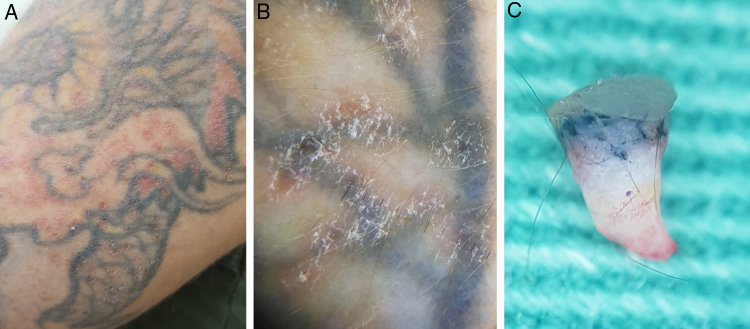
(A) Clinical appearance of the tattoo, with multiple discrete and diffuse erythematous papules, both on the tattoo and on healthy skin. (B) *In vivo* dermoscopy (×10 magnification) showing details of the papules with unspecific desquamation, without digitiform projections and red dots. (C) *Ex vivo* dermoscopy (×20 magnification) showing a slightly thickened upper portion the epidermis with discrete flat papillomatous projections, associated with the presence of pigment in the superficial and medium dermis.

**Figure 4:**
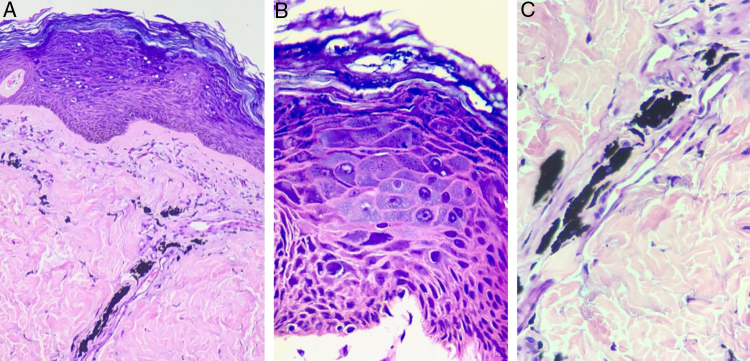
Histopathological exam of Patient 2, stained with hematoxylin & eosin. In (A) (×200 magnification) hyperkeratotic epidermis with the presence of balonized keratinocytes on the epidermal surface is observed. In the dermis, there are exogenous black pigment deposits around the vessels. In (B) (×400 magnification), nuclear polymorphism and hyperchromia are evident, in addition to the broad and basophilic cytoplasm of keratinocytes, characterizing the cytopathic effect of HPV. In (C) (×400 magnification), the black pigment of the tattoo deposited around the vessels is observed.

Reports of skin contamination from the tattooing process have increased in recent years, from infectious bacterial diseases such as atypical mycobacterioses and leprosy, to viral infections such as warts and molluscum contagiosum, and to diseases transmitted by sexually transmitted infection such as syphilis. Such an increase has stimulated a joint action of the United States Food and Drug Administration with the Centers for Disease Control and Prevention to revise the health surveillance criteria for tattoo centers. Another important step was the education of health professionals for the recognition and appropriate treatment of infections and cutaneous reactions in tattoos.^2^

Warts are frequent skin conditions caused by the human papillomavirus (HPV), a DNA virus of universal distribution.^7^ Dysfunction in the epithelial barrier due to trauma causes microscopic disruptions in the skin, allowing viral transmission. In the cases presented, there was damage to the skin barrier during tattooing, with possible inoculation of the virus during the process.^3^ Viral warts in the affected area may arise after an incubation period that generally ranges from three weeks to eight months.^7^ In some cases, the latency period is longer, and may reach ten years, making it difficult to relate the onset of the lesion to direct inoculation through the procedure. In these cases, it is proposed that the development of warts would depend on dysregulation of the immune system,^8^ since cell-mediated immunity plays an important role in the host's response to HPV.^7^ This can be evidenced in the second patient with HIV, who presented more extensive and persistent lesions.

In a review of wart implantation on tattoos, it was observed that the risk of acquiring a wart on black pigment is seven-fold greater than on colored pigment or on non-tattooed skin.^6^ In the first case here reported, the patient had no comorbidities and presented warts restricted to the tattoo, while the second patient presented lesions that did not respect the pigment linearity, including healthy skin.

Dermoscopy was of great value in the reported cases. Aspects classically present in common and anogenital warts were observed in the lesions on the tattoos presented here in the first case,^9^, ^10^ making possible the diagnosis of the HPV-induced lesion, even over the pigment. In the second case, it was harder to identify such dermatoscopic features, which were also rare in the clinical presentation. Sequencing for HPV in this case would be very worthwhile to better characterize if the lesion is a beta-papillomavirus, and whether the HPV of the condyloma acuminata is the same as that of the tattoo. However, it was not possible to carry out such analysis.

Another illustrative aspect of this study is the *ex vivo* dermatoscopy, where it was possible to identify both the epidermal alterations caused by HPV and the dermal changes produced by the presence of tattoo pigment. These findings were compatible with those identified in the histopathological examination.

The importance of the present study is its description of the clinical, dermoscopic, and histological aspects of HPV infection associated with tattoos, as well as the findings of *ex vivo* dermatoscopy similar to those identified in the histopathological examination. The authors emphasize the importance of the clinical suspicion of HPV infection, manifested by the appearance of warts after tattooing, and the need to adopt public health measures that make this procedure safer, in order to avoid future events.

## Financial support

None declared.

## Authors’ contribution

John Verrinder Veasey: Approval of the final version of the manuscript; conception and planning of the study; elaboration and writing of the manuscript; obtaining, analyzing, and interpreting the data; effective participation in research orientation; intellectual participation in propaedeutic and/or therapeutic conduct of the cases studied; critical review of the literature; critical review of the manuscript.

Ana Luisa Nasser Erthal: Conception and planning of the study; elaboration and writing of the manuscript; obtaining, analyzing, and interpreting the data; critical review of the literature; critical review of the manuscript.

Rute Facchini Lellis: Obtaining, analyzing, and interpreting the data; intellectual participation in propaedeutic and/or therapeutic conduct of the cases studied.

## Conflicts of interest

None declared.
